# A parameter-adaptive dynamic programming approach for inferring cophylogenies

**DOI:** 10.1186/1471-2105-11-S1-S60

**Published:** 2010-01-18

**Authors:** Daniel Merkle, Martin Middendorf, Nicolas Wieseke

**Affiliations:** 1 Department of Mathematics and Computer Science, University of Southern Denmark, Odense, Denmark; 2 Parallel Computing and Complex Systems Group, Department of Computer Science, University of Leipzig, Germany

## Abstract

**Background:**

Coevolutionary systems like hosts and their parasites are commonly used model systems for evolutionary studies. Inferring the coevolutionary history based on given phylogenies of both groups is often done by employing a set of possible types of events that happened during coevolution. Costs are assigned to the different types of events and a reconstruction of the common history with a minimal sum of event costs is sought.

**Results:**

This paper introduces a new algorithm and a corresponding tool called CoRe-PA, that can be used to infer the common history of coevolutionary systems. The proposed method utilizes an event-based concept for reconciliation analyses where the possible events are cospeciations, sortings, duplications, and (host) switches. All known event-based approaches so far assign costs to each type of cophylogenetic events in order to find a cost-minimal reconstruction. CoRe-PA uses a new parameter-adaptive approach, i.e., no costs have to be assigned to the coevolutionary events in advance. Several biological coevolutionary systems that have already been studied intensely in literature are used to show the performance of CoRe-PA.

**Conclusion:**

From a biological point of view reasonable cost values for event-based reconciliations can often be estimated only very roughly. CoRe-PA is very useful when it is difficult or impossible to assign exact cost values to different types of coevolutionary events in advance.

## Background

Due to the immense increase of available molecular data and the methodological improvements in computer science to handle this data, methods for analyzing the coevolution of large data sets of two groups of species become more and more sophisticated. Examples of such coevolutionary systems are hosts and their parasites, insect-plant relations, or symbiotic relationships. Different methods for reconstructing the common host parasite relations have been proposed in the literature (for an overview see, e.g., [1,2]). One common approach is to use an evolutionary model that describes the set of possible types of events that happened during coevolution, and to assign costs for the different types of events. The problem is then to find a reconstruction of the common history with a minimal sum of event costs.

Algorithms that employ this idea are called event-based methods [3]. Typically the four different types of events that are considered are cospeciation events, duplication events, sorting events and switching events (see [9]). The tools that are most commonly used in biological studies that use event-based methods for the analysis of coevolving species associations are TreeMap [4] and TreeFitter [5]. Notable are also Tarzan [6] and Jane [7], as they can handle more complex timing information about the phylogenetic trees than other methods. This is important because several recent studies of cophylogenetic relationships have shown that timing information can be very important for the correct interpretation of results from cophylogenetic analysis. Whereas these tools differ regarding several aspects, e.g., efficiency, the possibility to include timing information, or the availability of a graphical user interface, they all have in common that the event-based approach requires a cost assignment for the coevolutionary events in advance in order to compute a cost minimal reconstruction.

In this paper a new algorithm for event-based cophylogeny reconstruction and the corresponding tool called CoRe-PA are presented. The new method is based on a dynamic programming formulation for the cophylogenetic reconstruction problem and has significant new features compared to the current state-of-the-art methods TreeFitter, TreeMap, and Tarzan (compare also the paper [8] and the recently published tool Jane [7] where a dynamic formulation is used as well). Algorithm CoRe-PA can handle associations of parasites with multiple hosts, it includes the handling of divergence timing information. Unlike most other tools it can handle multifurcations in the input trees. It is suitable also for large phylogenetic trees due to a dynamic programming formulation for the reconstruction problem. Most notably however is the parameter-adaptive reconstruction approach of CoRe-PA. Unlike other event-based methods, in CoRe-PA no costs have to be assigned to the coevolutionary events. This is achieved by a careful definition of an underlying optimization criterion.

The paper is structured as follows. In the methods section a dynamic programming formulation for inferring cophylogenies is introduced. Furthermore the parameter-adaptive approach utilized in CoRe-PA is described and it is explained how randomized tests can be performed. In later sections several cophylogenetic systems are analyzed.

## Methods

### Basic definitions

**Figure 1:**
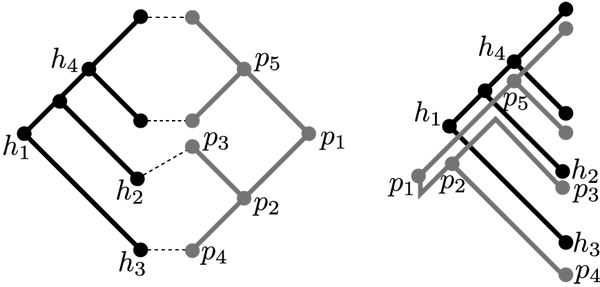
**Example for a coevolutionary system and a corresponding reconstruction**. Left: Example for a small coevolutionary system with four host species (leaf nodes in black tree) and four parasite species (leaf nodes in grey tree). Right: Example for a cophylogenetic reconstruction for the coevolutionary system. The three associations (*p*_3_, *h*_2_), (*p*_4_, *h*_3_), and (*p*_2_, *h*_1_) induce one cospeciation and one sorting event. The three associations (*p*_2_, *h*_1_), (*p*_5_, *h*_4_), and (*p*_1_, *h*_1_) induce one duplication and two sorting events. The reconstruction need two cospeciations, one duplication, and three sortings.

Let *H *and *P *be two phylogenetic trees. *H *and *P *will be called host tree, respectively, parasite tree. Let *φ *: *L*(*P*) × *L*(*H*) be a relation over the set of leaf nodes of the parasite tree and the leaf nodes of the host tree. *φ; *is used to describe known host-parasite interactions. A toy example for a cophylogenetic system of four hosts and four parasites and their associations is given in Figure 1 (left).

**Figure 2:**
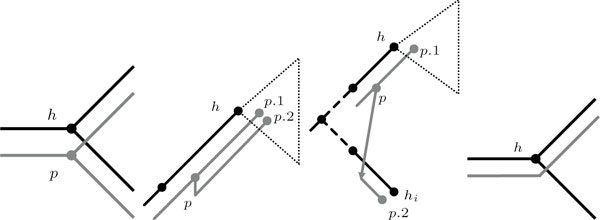
**Coevolutionary events**. From left to right: Cospeciation (node *p *associated with node *h*), duplication (both child nodes of *p *are associated with a node in the subtree of *H *with root *h*), switch (only one child node of *p *is associated with a node in the subtree of *H *with root *h*) and sorting. Host tree *H *is depicted black, parasite tree *P *is depicted grey.

In order to investigate whether there exists coevolution between hosts and their parasites, their common history is reconstructed from the phylogenies and the known current relationships. Typically, four different types of events are considered for the coevolutionary reconstruction of host-parasite systems: cospeciation events, duplication events, sorting events, and switching events. Cospeciation events refer to simultaneous speciations of host and parasite, duplication events are independent parasite speciations, sorting events correspond to lineage sorting (i.e., a parasite species that lives on a host species remains on only one of the resulting species after a host speciation), and switching events correspond to host shifts. As has been done by other authors (e.g., [3] and [9]) we consider a switch as a speciation of the parasite where one of the resulting species switches to another host. The four event types, that are also utilized in CoRe-PA are depicted in Figure 2.

We need the following definitions. If *p *is a node of a tree, then *p*.*i *denotes the *i*-th child node of *p*. The out-degree of node *p *is denoted with deg(*p*). An association of a parasite *p *∈ *P *to a host *h *∈ *H *is denoted as (*p*, *h*). A reconstruction *R *is the set of all associations of all parasites to nodes in the host tree, i.e., for each node *p *∈ *P *it exists an *h *∈ *H *such that (*p*, *h*) ∈ *R*. A reconstruction is valid if i) all parasite leaves are mapped to host leaves according to *φ*, ii) if node *p *is mapped to node *h*, then no descendant of *p *is associated with an ancestor of *h*, as this would induce an inconsistency, and iii) at least one child *p*.*i *of *p *has to be associated with an descendant of *h*. We do not consider the case of a speciation of the parasite *p *where both child species change to hosts that are outside of the subtree with root h because such events can not be traced back (many other studies also do not allow such events, e.g., [9]).

Based on a valid reconstruction *R*, the events implied by the associations in *R *can be inferred as follows. For all non-leaf nodes *p *∈ *P *the association of *p *and of all its children *p*.*i*, 1 ≤ *i *≤ deg(*p*), is considered. If for example, in the case of binary trees, the association (*p*, *h*) exists, and *p*.1 is mapped to one child of *h *and *p*.2 is mapped to the other child of *h*, then this implies either i) one cospeciation event, or ii) a duplication and two sorting events. This association triple technique has been used before in Tarzan and leads to an efficient reconstruction method (for details see [6]). A valid reconstruction for the coevolutionary system of Figure 1 (left) is depicted in Figure 1 (right). In the reconstruction the three associations (*p*_3_, *h*_2_), (*p*_4_, *h*_3_), and (*p*_2_, *h*_1_) induce one cospeciation and one sorting event (in general many different sets of events may be possible). The three associations (*p*_2_, *h*_1_), (*p*_5_, *h*_4_), and (*p*_1_, *h*_1_) induce one duplication and two sorting events. The depicted reconstruction requires two cospeciations, one duplication, and three sortings.

We will discuss divergence timing information and incompatible reconstruction only briefly in this article and refer to [9] and [6]. Considering again an association (*p*, *h*), where one child *p*.*i *is mapped to a node *h'*, and *h' *is not a descendant of *h*, then this implies (at least) one host switching event. A problem with switches in a reconstruction is that they induce a timing relation between the take-off site and the landing site. A consequence is that the occurrence of several switches in a valid reconstruction can lead to timing relations which are not possible. CoRe-PA includes more sophisticated methods for detecting these so-called incompatible (in contrast to compatible) reconstructions than, for example, Tarzan (for details how these incompatibilities can be resolved see [6]). However we will focus on the parameter-adaptive reconstruction approach in this article. Furthermore, we point out that CoRe-PA includes the same handling of divergence timing information as Tarzan, i.e., nodes can be labeled with divergence timing information and an association (*p*, *h*) is only allowed, if the timing information of *p *and *h *do not disallow this association.

### Dynamic programming approach

In the following a dynamic programming formulation (DP) for the reconstruction problem is given, which is a key component of CoRe-PA. We briefly discuss how the usage of divergence timing information is included, and explain details of runtime optimization techniques that are used. We omit a detailed discussion of how multifurcations and multiple-host parasites are handled (instead of resolving multifurcations by iterating over all possible binary subtrees, as done in Tarzan, non-binary cophylogenetic events where introduced, e.g., events that represents a composition of multiple duplications and cospeciations occurring consecutively taking into account the structure of multifurcating host and parasite nodes).

#### Initial DP formulation

The basic idea of the dynamic programming approach is to traverse the parasite tree *P *in a bottom-up manner. The cheapest cost *C*_*p*, *h *_for a node *p *of *P*, that is mapped on a node *h *of *H*, is stored in the dynamic programming table. If *p *is a leaf node, then the mapping for *p *is defined by the relation *φ *and induces no costs as no coevolutionary event occurs. In the recursive step of the dynamic programming we map all children *p*.1, ..., *p*.deg(*p*) of *p *to nodes in *H*. The mapping of the nodes *p*.*i *to nodes *h*_*i *_∈ *H *induces i) the recursively computed cost  for each association, plus ii) the cost from the cheapest set of events due to *p *being associated with *h*, and the nodes *p*.1, ..., *p*.deg(*p*) being associated with the corresponding *h*_*i*_. Note that there may exist several possibilities for this set of events to explain the given associations, and the cost-wise cheapest of those is taken. These costs are denoted by min(*E*(*h*, *h*_1_, ..., *h*_deg(*p*)_)). Let us consider again the binary example where *h*_1 _and *h*_2 _are children of *h *(i.e., *h*_1 _= *h*.1 and *h*_2 _= *h*.2, or *h*_1 _= *h*.2 and *h*_2 _= *h*.1). In this example min(*E*(*h*, *h*_1_, *h*_2_)) refers either to the costs for one cospeciation event or to the costs for one duplication and two sorting events. The dynamic programming formulation is as follows:(1)

For details on how min(*E*(*h*, *h*_1_, ..., *h*_deg(*p*)_)) is computed in the binary case see Algorithm 1.

**Algorithm 1**: Computing min(*E*(*h*, *h*_1_, *h*_2_)) in the binary case of Equation 1

**Input**: *h*, *h*_1_, *h*_2_, cospeciationCost, sortingCost, duplicationCost, hostswitchCost

**Output**: costs *E*

**1 ***E *← ∞;

**2 if ***h is not a descendant of h*_1 _*or h*_2 _**then**

**3**   ***S *** ← Compute number of sortings from *h *to *h*_1 _plus sortings from *h *to *h*_2_;

**4**   **if ***h*_1 _and *h*_2 _*are in the subtree with root h ***then**

**5**      *E *← *duplicationCost *+ (S * *sortingCost*);

**6**      **if ***h*_1 _and *h*_2 _*are in different subtrees with root h*.1 *and h*.2 **then**

**7**         *E *← min(*E*, *cospeciationCost *+ ((*S *- 2) * *sortingCost*);

**8**      **end**

**9**   **end**

**10**   **if ***either h*_1_*or h*_2_*is in subtree with root h ***then**

**11**      *E *← *hostswitchCost *+ (S * *sortingCost*);

**12**   **end**

13 end

**14 return ***E*;

#### Inclusion of divergence timing information

Similar to the approach in [6], algorithm CoRe-PA allows assigning intervals of time zones to the nodes in one of the trees, e.g., the parasite tree. The nodes in the other tree, e.g., the host tree, have to be assigned to a single time zone. The reason for this is that the reconstruction problem becomes much more complex when nodes in both trees are assigned to time zone intervals [6]. For each possible association (*p*, *h*) we define a value *Z*_*p*, *h*_. The value of *Z*_*p*, *h *_is 0 if the association is valid with respect to the timing information, and it is ∞ otherwise. For the revised DP formulation we add the value *Z*_*p*, *h *_in the recursion step of Equation 1.

#### Optimization

A direct implementation of the DP formulation, as given in Equation 1, would not perform very well, as all possible combinations of all possible associations of nodes *p*.*i *to nodes *h*_*i *_would be considered in order to compute *C*_*p*, *h*_, i.e., any of the *n*^*deg*(*p*) ^combinations of choosing *deg*(*p*) hosts out of the n nodes in the host phylogeny have to be considered. Therefore several improvements are included into the implementation of CoRe-PA. The most important improvement reduces the number of combinations of associations that have to be considered significantly as described in the following. If the costs for *C*_*p*, *h *_are computed according to Equation 1, all possible mappings of each *p*.*i *to all *h *∈ *H *are considered. Let us assume two possibilities for mappings of *p*.*i*, namely *p*.*i *being mapped to *h' *and *p*.*i *being mapped to *h"*. Let us further assume that *h' *and *h" *are both in a subtree of *H *that has a child of *h *as a root node. As we know the values of *C*_*p*.*i*, *h' *_and *C*_*p*.*i*, *h*″ _(due to the recursive approach) and as the number of sorting events induced by the pair of associations (*p*, *h*) and (*p*.*i*, *h'*) (respectively (*p*, *h*) and (*p*.*i*, *h"*)) is known, one of the associations (either (*p*.*i*, *h'*) or (*p*.*i*, *h"*)) will dominate the other (unless the costs are equal). This is true for every pair of host nodes that occur in the same subtree of *H *that have a child of *h *as root node. Therefore, only the association that induces the smallest cost in such a subtree must be considered and the number of combinations to be considered in the recursive approach is reduced significantly. This is not only true for all these subtrees, but also for the set of all other nodes that are neither *h *itself nor in one of the just described subtrees. For the binary case pseudocode is given in Algorithm 2.

**Algorithm 2**: Computing *C*_*p*, *h *_in the binary case of Equation 1

**Input**: parasite *p*, host *h*, *C*_*p*.1, *h'*_, *C*_*p*.2, *h"*_, for all *h'*, *h" *∈ *H*

**Output**: *C*_*p*, *h*_

**1 if ***p *∈ *L*(*P*) **then**

**2**   **return **((*p*, *h*) ∈ *φ*)?0:∞;

3 end

**4 ***T *← 4-partition of *H *specified by {nodes of subtree *h*.1, nodes of subtree *h*.2, {*h*}, remaining nodes};

**5 ** ← for each *T*^*j *^∈ *T *choose the *h' *∈ *T *^*j *^with min(*C*_*p*.1, *h' *_+ sorting costs from *h *to *h'*);

**6 ** ← for each T ^*j *^∈ *T *choose the *h" *∈ *T*^*j *^with min(*C*_*p*.2, *h" *_+ sorting costs from *h *to *h''*);

**7 ***C*_*p*, *h *_← ∞;

**8 foreach ***h*_1 _*from ***do**

**9**   **foreach ***h*_2 _*from ***do**

**10**      *C*_*p*, *h *_← min(*C*_*p*, *h*_,  +  + min(*E*(*h*, *h*_1_, *h*_2_)));

**11**   **end**

12 end

**13 return ***C*_*p*, *h*_;

In addition to this dominance-based optimization CoRe-PA utilize tables of precomputed event costs. Assume that an arbitrary parasite node *p *is being mapped on *h *and a child *p*.*i *of *p *is being mapped on *h'*. A certain set of events that have to occur can be precomputed independent from the specific choice of *p *and *p*.*i*: for example, if *h' *is a descendant of *h*, the number of sorting events can be computed; in other cases host switches can be inferred beforehand. In order to perform such precomputations, it is assumed that each possible *h *and *h' *for the mapping of an arbitrary *p *and the child node *p*.*i *is considered. Also in the case that divergence timing information is used, the best take-off and landing sites can be precomputed in the same manner.

Let *n *be the maximal number of nodes in the host or in the parasite tree. It is not difficult to see, that computing a reconstruction with CoRe-PA runs in order of *O*(*n*^3^), if the maximal degree of the nodes in the trees is assumed to be constant.

### Parameter-adaptive cophylogenetic reconstruction

Several optimization criteria have been investigated in the literature that utilize event-based cophylogenetic reconstruction methods. Examples include the minimization of overall reconstruction costs or the maximization of the number of cospeciations. But all methods are strongly dependant on a good estimation of the cost vector, that assigns costs to the events. Often cospeciation costs are considered to be small (for example ≤ 0), and duplication and host switch costs are usually assumed to be high. However, from a biological point of view, the exact values for these costs are basically unknown. In [3] an inspiring comment is given: "*If each event is associated with a cost that is inversely related to the likelihood of the event (the more likely the event, the smaller the cost) then the most parsimonious reconstruction will also, in some sense, be the most likely explanation of the observed data*.". This comment nicely reflects the underlying idea of the parameter-adaptive approach of CoRe-PA, that will be described in the following. Unlike other methods CoRe-PA does not require any restrictions on the cost values. However, for the parameter-adaptive approach we assume all event costs are between 0 and 1 (If they are larger this can be achieved by multiplication with a suitable factor, as only the ratio between the event costs have an effect on the reconstruction and not the values themselves). Let  = (*c*_1_, ..., *c*_*m*_) be the cost vector for the *m *possible events. Based on this cost setting it is expected that the event indexed by *i *occurs with probability(2)

i.e., the probability for a certain event is the normalized value of the reciprocal event cost. This ensures that also the ratio between the probabilities of two events are inversely proportional to the ratio between the corresponding cost values. Note that negative cost values can not be considered in this parameter-adaptive approach, as negative probability values can not be interpreted reasonably. However, from a parsimony perspective negative cost values are questionable anyway (see [3]).

Based on the cost vector a cost-minimal reconstruction is inferred using the DP formulation as given above; this in turn leads to relative event frequencies *r*_*i *_of the events, based on the computed reconstruction. Assume that cost vector  is used to determine a reconstruction. The obvious method to determine how good the reconstruction and the cost vector fit, is based on the sum of the differences of the probabilities *p*_*i *_and the corresponding relative event frequencies *r*_*i *_of the reconstruction. Formally,(3)

By using  as an optimization criteria, a cost vector  is sought such that  is minimized. The value  can be interpreted as a quantification of how unlikely a reconstruction is. Furthermore, if, based on some significance test, there is a strong support for coevolution, but the corresponding  is very high, then the support for the coevolutionary signal has still to be questioned.

The parameter-adaptive approach reduces the parameterized cophylogenetic reconstruction problem to a parameter-adaptive optimization problem. Of course, many sophisticated methods are known for finding a good vector , like meta-heuristics [10] or utilizing the concept of a simplex (like in the Nelder-Mead downhill simplex method [11]). In order to be able to present a reasonable statistical analysis of the parameter-adaptive component of CoRe-PA and not to be biased by an underlying optimization method, we present only results that are based on randomly chosen (uniform distribution) cost vectors (although the Nelder-Mead simplex method is already included in CoRe-PA).

### Randomized tests in CoRe-PA

In order to evaluate whether the number of different phylogenetic events of a reconstruction indicates significant coevolution, different randomization tests can be used (see, e.g., [12]). The idea of these tests is to create reconstructions for scenarios where part of the problem instance is randomly changed, e.g., the hosts and parasite associations can be changed randomly. Then the number of events in the reconstructions for the random scenarios can be compared to the reconstruction for the original host parasite scenario. Different opinions have been stated in the literature about what part of the host-parasite data should be randomized when creating random instances for a significance test. Some possibilities are to randomize the parasite tree, the host tree, both trees, or the associations between host and parasites (see [12]). It is important that the random instances are biologically plausible because otherwise the significance results that can be obtained with the tests are biologically useless. Therefore, different methods have been proposed how the random instances should be generated (see [13] for an overview). One randomization test that is integrated in TreeMap is the most often used test in literature on host parasite coevolution (see, e.g., [14]). The test asks whether the maximum proportion of cospeciating nodes inferred is greater than the maximum proportion that can be inferred when one of the phylogenies is randomized. TreeMap allows to randomize either one tree (the host or the parasite tree) or both trees. All these possibilities have been used in the literature.

In [12] the proper use of randomization methods in order to analyze, whether the fit between hosts and parasites can be explained by coevolution, is discussed. It was argued that for a corresponding test it is not appropriate to make random changes in the host or parasite tree. Instead it was proposed to keep the phylogenies of the hosts and the parasites as well as the number of associations. Only the associations between the hosts and parasites should be randomized. This method has been used, e.g., in [14]. For many host parasite systems it can be observed that the number of different parasite species on one host species is small. For such a system it might not be biologically meaningful if a random association between hosts and parasites is created by assigning each parasite a random host with equal probability. Therefore we propose here that random associations should be created that keep the character of the host parasite assignment in the following sense. The number of hosts that have *k *parasite species should be the same in the original host parasite system and the random instance for all integers *k*. We call this a character preserving association. All the discussed methods are included in CoRe-PA. In the case that random trees have to be generated, the well known *β*-splitting model [13] is employed. The *β*-splitting model includes the Markov model and the PDA model as special cases. The method for randomizing the parasite tree (resp. the host tree and both trees) is denoted by RND-parasite (resp. RND-host and RND-both); the character preserving association is denoted by RND-assoc.

## Results and discussion

Six biological coevolutionary systems that have already been studied intensely in the literature are used as test examples in this study. Note that in coevolutionary systems multifurcations are often resolved artificially into bifurcations, although there are clear indications that the support for this method based on the biological data is very weak. Furthermore, if not stated otherwise, the data sets from the literature do not contain multi-host parasites, although there is sometimes support for this in the underlying data. These restrictions are necessary in order to be able to use standard tools for cophylogenetic reconstruction; CoRe-PA would not require these restrictions. When generating random trees with the *β*-splitting model, we always use *β *= -1 as suggested in [13]. Note that all reconstructions in this section, which are suggested by CoRe-PA, are compatible.

### Biological data sets

The test systems are one system - denoted by *S*_1 _- of gophers hosts and lice parasites (see Figures 11 and 13 in [9]), two systems - denoted by *S*_2-*ML *_and *S*_2-*MP *_- of *Pelecanicform *bird hosts and *Pectinopygus *lice parasites (see Figure 2, 4, and 5 in [15]), one system - denoted by *S*_3 _- of *Hystricognathi *rodents and pinworm parasites (see Figure 6.5 in [16]), one system - denoted by *S*_4 _- of seabirds and their chewing lice (see Figure 12.4 in [17]) and a recently presented system - denoted by *S*_5 _- of *Microbotyrum *funghi and their *Caryophyllaceae *hosts that includes multihost parasites (see Figure 4 in [18]).

### Parameterized reconstruction of random trees

**Figure 3:**
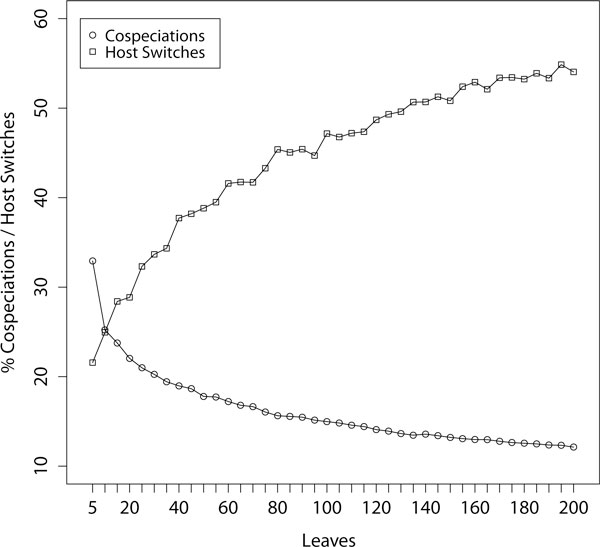
**Development of switching and cospeciation event frequencies**. Mean frequency of switch and cospeciation events based on random tree pairs with 5, 10, ..., 200 leaf nodes. Fixed costs for cospeciation, sorting, duplication, and host switches are *co *= -2, *so *= 1, *du *= 2, and *hs *= 4.

A problem with inferring cophylogenetic reconstruction based on a (standard) cost vector is that the frequencies of certain events strongly depend on the size of the input data set. To investigate this, we created 100 random tree pairs with random associations for 5, 10, ..., 200 leaf nodes (all together 4000 tree pairs). A fixed cost vector was used with cost settings for cospeciation, sorting, duplication, and host switches being *co *= -2, *so *= 1, *du *= 2, and *hs *= 4. Note that in standard cost vectors used in literature, the switching event has usually lower costs (*hs *= 2). The 40 mean values for the frequencies of the number of host switches and for the number of cospeciations, based on the 40 sets of 100 random tree pairs, are depicted in Figure 3. The results clearly indicate that, even though higher switching costs were used, host switches become more and more likely when larger phylogenetic trees are used (respectively cospeciations become more and more unlikely). This indicates that when using standard cost vectors the frequency of switching and cospeciation events in a cost minimal reconstructions depends heavily on the size of the phylogenetic trees. So these parameter values seem to be not very realistic for coevolutionary analysis.

### Parameter-adaptive reconstruction

When using the parameter-adaptive approach of CoRe-PA, 100000 cost-minimal reconstructions are computed based on randomly chosen cost vectors. The reconstruction with the smallest value for  (compare Equation 3) is the reconstruction suggested by CoRe-PA. When employing one of the randomization methods RND-{host, parasite, both, assoc} for each randomized instance 100000 cost-minimal reconstructions are computed based on randomly chosen cost vectors, and the resulting value  refers to the best of these.

**Figure 4:**
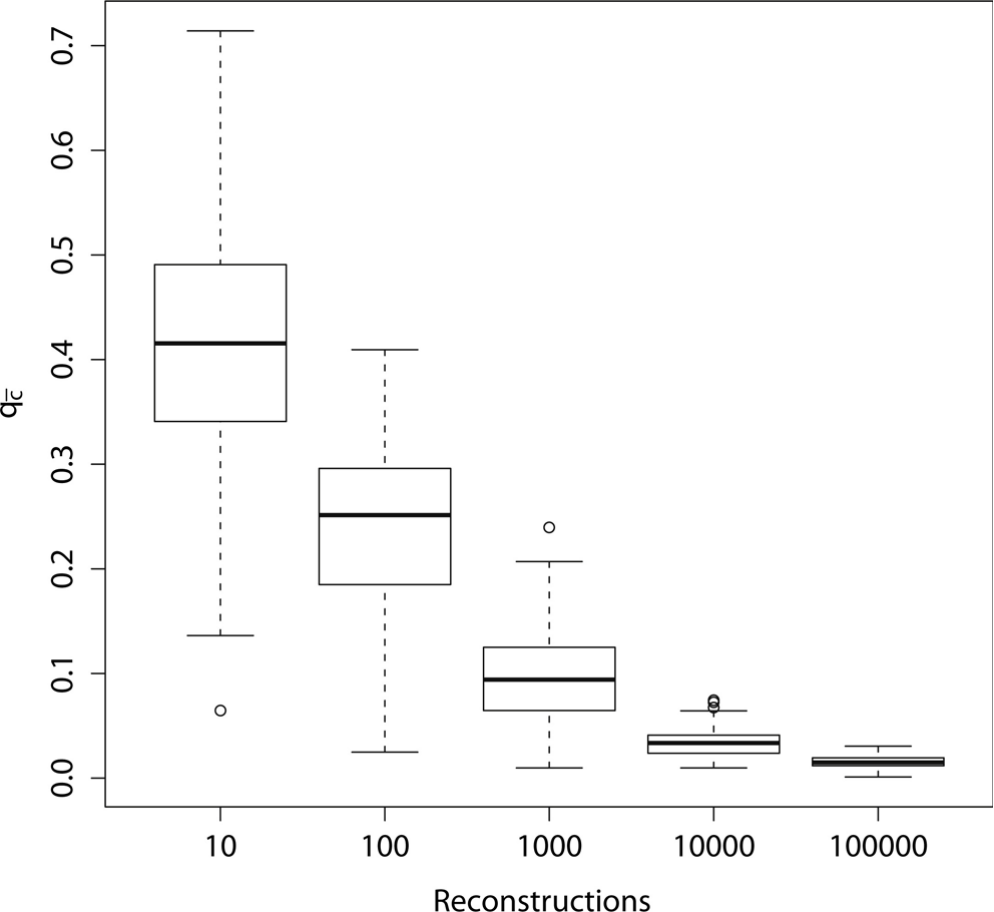
**Convergence behavior of **. Convergence behavior based on  for CoRe-PA on data set *S*_1 _when searching for the best cost vector. Depicted are box plots for  for 100 independent test runs after 10, 100, 1000, 10000, 100000 cost vectors have been chosen randomly.

In Figure 4 the convergence behavior of CoRe-PA is depicted for system *S*_1_. Given are box plots of  based on 100 test runs that were stopped after 10, 100, 1000, 10000, and 100000 cost vectors have been chosen randomly in each run. The results indicate that the algorithm is in a nearly converged state after 100000 randomly chosen cost vectors were used.

**Figure 5:**
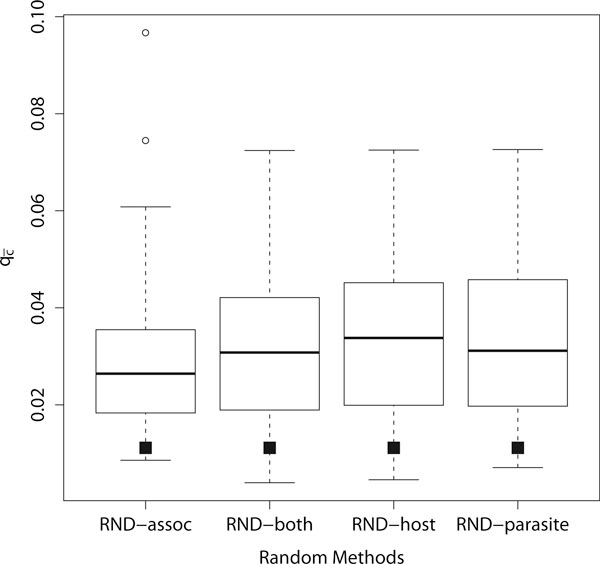
**Distribution of  using different randomization methods**. Randomization methods RND-{assoc, both, host, parasite} on system *S*_4_. For each box plot 100 random instances were created and  was computed based on 100000 reconstructions for each instance. Black squares indicate the outcome of CoRe-PA for the unmodified test instance.

**Figure 6:**
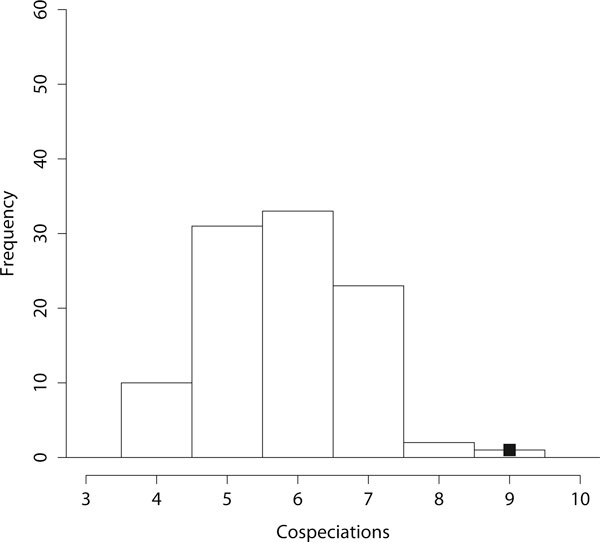
**Distribution of cospeciation event frequency for system *S*_4_**. Histogram for the number of cospeciations for system *S*_4 _when using randomization method RND-assoc. Based on the original instance CoRe-PA suggested a reconstruction with 9 cospeciations. The black square indicates the outcome of CoRe-PA for the unmodified test instance.

Results for the four different randomization methods are given in Figure 5 for system *S*_4_. Depicted are the box plots for  (100 randomized test instances were created based on the methods RND-{host, parasite, both, assoc}). It can be seen that the method of randomization has only a small influence on the overall result of , and that  is significantly smaller for the original instance compared to randomized instances. In the rest of this section we only employ the method RND-assoc (the results for the other randomization methods were very similar). The frequency of the number of cospeciations that occurred in the randomized instances for *S*_4 _(method RND-assoc) are depicted in the histogram in Figure 6. This figure clearly indicates the strong support for coevolution, as only a very small number of reconstructions had the same number of cospeciations as the reconstruction suggested by CoRe-PA and no reconstruction had more cospeciations.

**Figure 7:**
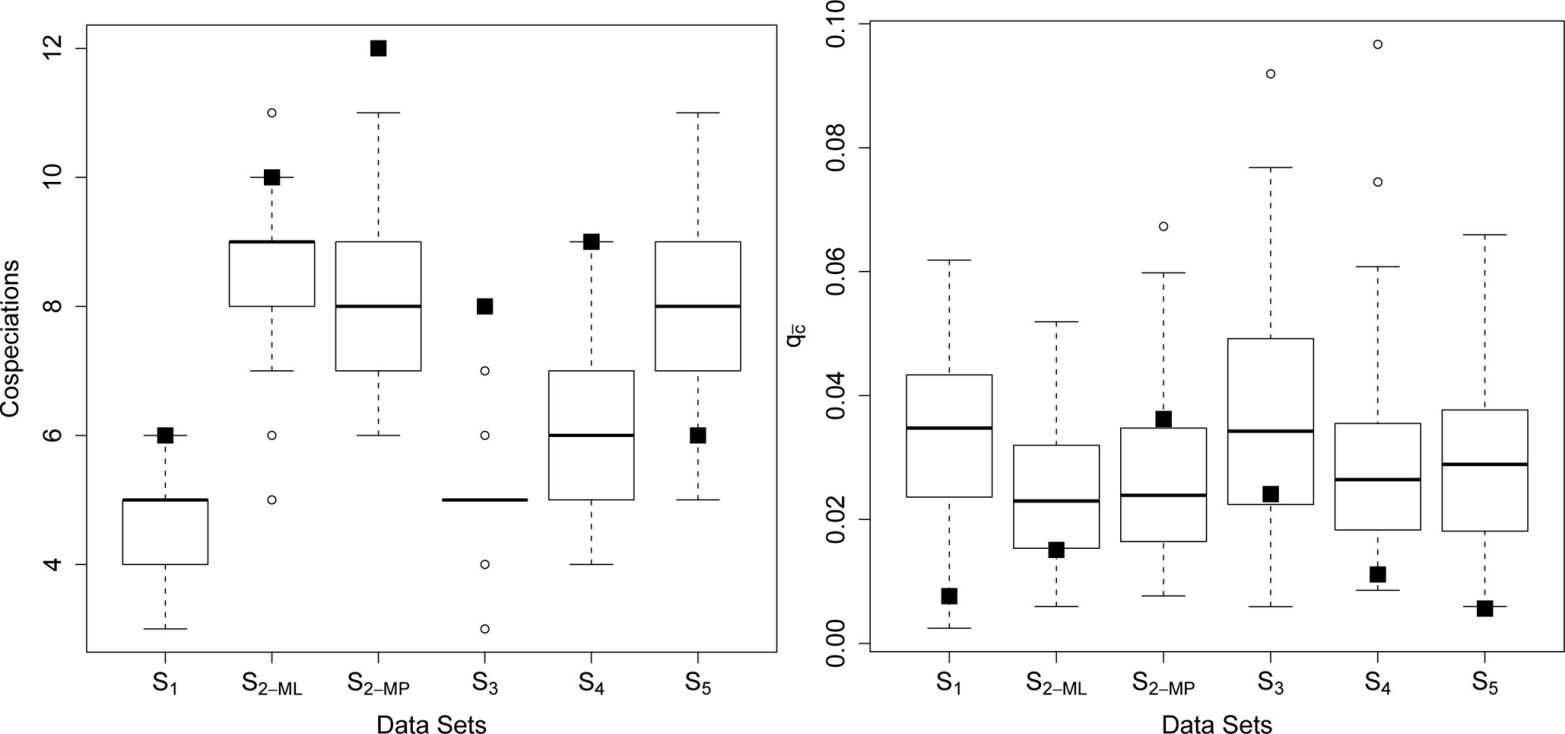
**Distributions of cospeciation event frequency and ****for systems *S*_1_, ..., *S*_5_**. Box plots for the number of cospeciations (left) and  (right) based on 100 randomized test instances (method RND-assoc) for systems *S*_1_, ..., *S*_5_. Black squares indicate the corresponding value for the solution suggested by CoRe-PA.

Table 1 shows the results of CoRe-PA for all six coevolutionary systems. For each system we give the number of events, the best cost vector, and the value for  for the solution having the smallest value for . For each system 100 randomized instances were created by using method RND-assoc; the column *p*_*co*,>_/*p*_*co*,≥ _(respectively *p*_*qu*_) denotes the probability, that a randomized instance lead to reconstructions with an equal number or more coevolutionary events (respectively to reconstructions with a smaller ). Figure 7 (left, respectively right) depicts the box plots for the number of cospeciations (respectively for ) based on the 100 randomized instances, and the number of cospeciations (respectively ) for the reconstruction suggested by CoRe-PA for the unmodified test instance (indicated by the black square).

**Table 1:** Reconstruction results for systems *S*_1_, ..., *S*_5_.

System	event frequency	best cost vector		*p*_*co*,>_/*p*_*co*,≥_	*p*_*qu*_
*S*_1_	(6, 5, 2, 1)	(0.166, 0.198, 0.512, 0.987)	0.008	0.00/0.13	0.04
*S*_2-*ML*_	(10, 20, 5, 2)	(0.226, 0.114, 0.457, 0.989)	0.015	0.04/0.13	0.24
*S*_2-*MP*_	(12, 18, 5, 0)	(0.007, 0.005, 0.018, 0.882)	0.036	0.00/0.00	0.78
*S*_3_	(8, 15, 3, 1)	(0.095, 0.053, 0.268, 0.738)	0.024	0.00/0.00	0.28
*S*_4_	(9, 11, 3, 1)	(0.077, 0.061, 0.224, 0.667)	0.011	0.01/0.03	0.05
*S*_5_	(6, 32, 9, 4)	(0.388, 0.072, 0.252, 0.587)	0.006	0.87/0.98	0.00

The event order for the vectors in column 2 (absolute event frequency) and column 3 (best cost vector) is (cospeciation, sorting, duplication, host switch).  as in Equation 3. p*co*,> (respectively p*co*,≥): probability that a reconstruction based on a randomized instance leads to more (respectively, an equal number or more) cospeciations. p*qu*: probability that a randomized reconstruction leads to a smaller value of . In all test runs randomization method RND-assoc was used.

The results give a strong indication for a coevolutionary history of systems *S*_1 _and *S*_4 _with respect to the number of cospeciations. As  is very small for these systems this outcome should be interpreted as a clear sign of coevolution. Systems *S*_2-*ML*_, *S*_2-*MP*_, and *S*_3 _also show a strong evidence for coevolution based on *p*_*co*,≥_, but the support for this (compare *p*_*qu*_) is only reasonably good for *S*_2-*ML *_and *S*_3_, and bad for *S*_2-*MP *_(*p*_*qu *_= 0.78). The values for system *S*_5 _should be interpreted as a clear sign of no coevolution (*p*_*co*,≥ _= 0.98) with a strong support for this result based on *p*_*qu *_= 0.00. Note that the extensive studies in the literature [16,18] for systems *S*_2-*ML*_, *S*_2-*MP*_, and *S*_5 _also do not conclude that there is a clear coevolutionary signal, and the tools used showed partially contradicting results.

Although a detailed discussion of the reconstructions is not possible in this paper, we want to point out that for systems *S*_4 _(respectively *S*_1_, *S*_2-*ML*_, and *S*_2-*MP*_) the best reconstruction that was obtained by CoRe-PA is identical (or very similar) to the reconstructions that are given in the literature. But different from the results in the literature the reconstructions obtained by CoRe-PA did not assume any predefined costs for the coevolutionary events.

It is also noteworthy that in all systems, except *S*_1_, CoRe-PA obtained higher cost values for cospeciation events than for sorting events, which is contrary to standard cost vectors used in the literature. As expected the switching event had the highest cost values.

## Conclusion

We have introduced a new algorithm and a corresponding tool called CoRe-PA for parameter-adaptive cophylogenetic analysis. Different from other event-based reconstruction methods CoRe-PA does not require any cost settings for the considered cophylogenetic events in advance, but seeks for the cheapest reconstruction in which the used costs are inversely related to the relative frequency of the corresponding event. The quality of the reconstructions obtained with CoRe-PA was analyzed experimentally on six coevolutionary systems. The results show that CoRe-PA is very useful when it is difficult or impossible to assign exact cost values to different types of coevolutionary events in advance.

## Competing interests

The authors declare that they have no competing interests.

## Authors' contributions

All authors made substantive intellectual contributions to the published study. MM initiated this study. DM conceived the study and wrote the draft of this paper. NW developed, implemented, and tested the methods. All authors improved the draft version, and approved the final manuscript.
